# Investigating the Cellular Transcriptomic Response Induced by the Makona Variant of Ebola Virus in Differentiated THP-1 Cells

**DOI:** 10.3390/v11111023

**Published:** 2019-11-04

**Authors:** Andrew Bosworth, Stuart D. Dowall, Stuart Armstrong, Xuan Liu, Xiaofeng Dong, Christine B. Bruce, Lisa F. P. Ng, Miles W. Carroll, Roger Hewson, Julian A. Hiscox

**Affiliations:** 1 Public Health England, Manor Farm Road, Porton Down, Salisbury SP4 OJG, UK; Andrew.Bosworth@phe.gov.uk (A.B.); Miles.carroll@phe.gov.uk (M.W.C.); 2 Health Protection Research Unit in Emerging and Zoonotic Infections, National Institute for Health Research, Liverpool L3 5RF, UK; 3 Clinical Virology, Regional Public Health Laboratory, Public Health England, Bordeseley Green East, Birmingham B9 5SS, UK; 4 Institute of Infection and Global Health, University of Liverpool, Liverpool L3 5RF, UK; 5 Centre for Genomics Research, University of Liverpool, Liverpool L3 5RF, UK; 6 Singapore Immunology Network, A*Star, Biopolis, Singapore 138648, Singapore

**Keywords:** Ebola virus, Makona, West Africa, transcriptomics, proteomics

## Abstract

Recent studies have shown that transcriptomic analysis of blood samples taken from patients with acute Ebola virus disease (EVD) during the 2013–2016 West African outbreak was suggestive that a severe inflammatory response took place in acutely ill patients. The significant knowledge gained from studying the Makona variant, a cause of the largest known EVD outbreak, may be applicable to other species of ebolavirus, and other variants of the Ebola virus (EBOV) species. To investigate the ability of Makona to initiate an inflammatory response in human macrophages and characterise the host response in a similar manner to previously characterised EBOV variants, the human monocytic cell line THP-1 was differentiated into macrophage-like cells and infected with Makona. RNA-Seq and quantitative proteomics were used to identify and quantify host mRNA and protein abundance during infection. Data from infection with Reston virus (RESTV) were used as comparators to investigate changes that may be specific to, or enhanced in, Makona infection in relation to a less pathogenic species of ebolavirus.. This study found demonstrable induction of the inflammatory response, and increase in the activation state of THP-1 macrophages infected with Makona. NFκB and inflammation-associated transcripts displayed significant changes in abundance, reflective of what was observed in human patients during the 2013–2016 EBOV outbreak in West Africa, and demonstrated that transcriptomic changes found in Makona-infected cells were similar to that observed in Reston virus infection and that have been described in previous studies of other variants of EBOV.

## 1. Introduction

In the wake of the unexpected emergence of Ebola virus (EBOV) in West Africa [1,2], there has been renewed interest in the fundamental biology of EBOV. Like any virus, EBOV is an obligate intracellular parasite, and as such, is critically dependent on the host cell. 

Advances in next-generation sequencing have allowed the viral lifecycle to be studied at great depth in vitro [3,4], in animal models [5,6], and, most recently, in patients [7]. These studies identified the critical roles of host signalling pathways in the lifecycle of EBOV [4,8,9,10,11]. Complex virus–host interactions take place during EBOV infection [12,13,14], particularly the interactions of viral proteins (VP) conferring virulence: VP24 [15,16,17], VP35 [18,19], and those factors essential for virus lifecycle, such as VP30 [8]. Initial infection of macrophages and dendritic cells leads to widespread circulation and virus dissemination [20,21]. Infection of dendritic cells results in a reduction in pro-inflammatory cytokines and T-cell stimulatory receptors [15,20], a process that is postulated to have an important role in widespread lymphocyte apoptosis [22,23]. In contrast, infection of macrophages results in a pro-inflammatory phenotype, rapidly increasing levels of tumour necrosis factor (TNF), stimulating immune responses, bystander apoptosis, vascular leakage, and tissue re-modelling [20,22,24,25].

Studies of the macrophage transcriptome during infection with the Makona variant of EBOV have not yet been reported. There remains a question of whether Makona infection induces cellular changes in macrophage-like cells that may be distinct from other variants of EBOV. Macrophages have an important role to play in the spread of EBOV and the pathology of Ebola virus disease (EVD) [20,25,26,27]. To define the molecular events that take place during infection of macrophages with the Makona variant, differentiated THP-1 macrophage-like cells were used. The THP-1 cell line was derived from neoplastic monocytes, and can be differentiated into macrophage-like cells by treating with low concentrations of a protein kinase C (PKC) activator [28]. After resting, these cells express surface proteins and have morphology similar to activated macrophages [28,29,30]. As macrophage infection is so critical in the pathogenesis of EVD, a model of this cell type was selected for study in order to identify transcriptional changes that may contribute to successful viral infection and facilitate ongoing spread of the virus. Selection of a model cell line that was more representative of cells infected during the early stages of viral infection of man means meaningful data on the effects of Makona on a pathogenically relevant cell type has been collected. This study aimed to identify features of macrophage infection with the Makona variant that have not yet been described for this variant of EBOV. Through comparison with Reston virus (RESTV) infection, this study attempted to identify transcriptomic changes induced by Makona that may be important in disease pathogenesis. A recent comparative study of Makona with the Ecran (Mayinga) variant of EBOV in the A549 model cell line found small changes in the cellular inflammatory response [4]. THP-1 cells are recognised as a useful cell model to study inflammatory response and immune cell activation, and represent a cell type that is viewed as the primary target of ebolaviruses upon host entry.

Reston virus (RESTV) is a distinct species belonging to the ebolavirus genus, and has been reported to infect humans but not cause Ebola virus disease (EVD). RESTV causes pathology that is similar to EVD in non-human primate models of infection. Cell culture studies have also identified differences in the host response between EBOV compared to RESTV [3]. It is hypothesised that differences in the host response may be identified through comparison of transcriptional and proteomic changes during infection with Makona and RESTV, which may help to highlight transcriptional changes that are associated with different disease profiles caused in man. 

In this study, transcriptomics and quantitative proteomics were used to contrast and compare the host response in differentiated THP-1 cells infected with either Makona or RESTV, allowing the patterns of transcription to be investigated. Pathways and transcriptional regulation were highlighted that changed significantly during infection with Makona. Furthermore, comparison with RESTV infection allowed the identification of pathway activation conserved across pathogenically diverse ebolavirus species. The work presented here demonstrates a significant increase in the abundance of transcripts associated with the inflammatory response, which may be critical in the lifecycle of ebolaviruses. 

## 2. Materials and Methods

### 2.1. Mammalian Cell Culture and Viruses

THP-1 (derived from human monocytes) and Vero E6 (derived from African green monkey kidney) cell lines were obtained from the European Collection of Cell Cultures (ECACC) maintained by Public Health England. Cultures were authenticated by sequencing and tested to ensure they were both endotoxin- and mycoplasma-free prior to use. A near clinical isolate (passage 1) of Makona, obtained from the European Mobile Laboratory as part of the response to the 2014 West African outbreak and designated H. sapiens-wt/GIN/2014/Makona-Gueckedou-C05 [1], was amplified in Vero E6 cells to create the stock for this work. RESTV Pennsylvania variant M. fascicularis-wt/USA/1989/Pennsylvania [31] was cultured from archival stocks originating from the 1989 outbreak in the United States on Vero E6 cells. Both viruses used in this study were quantified by TCID50 by the Reed and Muench method [32] and tested to ensure virus stocks were both endotoxin- and mycoplasma-free. Terminally differentiated THP-1 cells were generated through 24 h treatment with 5 ng phorbol 12-myristate 13-acetate (PMA) (Sigma-Aldrich, London, United Kingdom), followed by media change to complete growth media and resting for 72 h prior to infection [28]. Differentiated THP-1 cells were infected with EBOV-Makona or RESTV at an MOI of 5 at Containment Level 4, and cellular RNA was purified at 0, 6, 24, 48, or 72 h post-infection using the Qiagen RNEasy Mini Kit, 0, 24, 48, or 72 h with the Qiagen AllPrep DNA and Protein extraction kit for cellular fractions for proteomic and transcriptomic analysis or the QIAmp Viral RNA Kit (Qiagen, London, United Kingdom) for cell-free viral RNA. 

### 2.2. qRT-PCR Analysis of Viral Load

RNA was extracted with the QIAmp Viral RNA Kit (Qiagen) and RNA yields were measured with a Nanodrop 1000 (Thermo Scientific). Two separate assays were developed and validated, one for RESTV and one for EBOV, with primers and minor-groove binder probe sequences selected from previously described assays [33], to quantify the relative abundance of the EBOV and RESTV genome. The reaction was set up with 900 nM of forward and reverse primers, 250 nM probe, 1× TaqMan Fast Virus Mastermix (Life Technologies, London, United Kingdom), and tested using the ABi 7500 (Life Technologies) at a thermal sequence of 50 °C for 5 min, 95 °C for 20 s followed by 40 cycles of 95 °C for 3 s and 60 °C for 30 s. Samples were tested in triplicate and results analysed at a threshold value of 0.2. For quantification of viral genome copies, a synthetic RNA oligo was designed and developed (Integrated DNA Technologies, Germany). Concentrations were validated by qRT-PCR and Qubit Analyzer (Thermo Scientific) and a dilution range from 10^9^ to 10^−1^ was used in duplicate.

### 2.3. RT2 Profiler Array Cards

RT2 Profiler Arrays (Qiagen) were used for cytokine profiling by qRT-PCR. The RT2 Antiviral Genes array card was selected for focusing on genes involved in viral infection and includes 84 genes of interest, housekeepers, and controls. Biological duplicate samples derived from THP-1 cells infected with EBOV or RESTV for 6, 24, 48, or 72 h were prepared, as were biological duplicates of mock-infected conditions for each cell line. RNA was treated with an Ambion Turbo DNA-free Kit and purified using the RNEasy MinElute purification kit (Qiagen). DNA-free RNA were added to RT2 Genomic Elimination Buffer (Qiagen) and treated for 10 min at 42 °C. RT2 Reverse Transcription (Qiagen) kit components were prepared in accordance with manufacturer instructions and mixed with the genomic elimination preparation. Reverse transcription was performed at 42 °C for 15 min, and reaction stopped by heating to 95 °C for 5 min prior to cooling on ice. The array cards were tested on the ABI 7500 (Applied Biosystems) using Qiagen RT2 SYBR Green Mastermix. Gene expression analysis was performed using the ΔΔCT method to determine fold change in relative gene expression, comparing each infected condition to mock-infected, of the relevant cell line. Housekeepers used in this calculation included GAPDH, HPRT, Beta-Actin, Alpha-Tubulin, and B2M. 

### 2.4. RNA-Seq

The RNA samples were DNase treated using an Ambion Turbo DNA-free Kit, and subsequently purified using Ampure XP beads. Then, 2 μg of the DNase treated RNA was put through a Ribozero treatment using the Epicentre Ribo-Zero Gold Kit (Human/Rat/Mouse) and purified again with Ampure XP beads. Successful depletion was then quality tested using Qubit and Agilent 2100 Bioanalyzer, and all of the depleted RNA was used as input material for the ScriptSeq v2 RNA-Seq Library Preparation protocol. Two biological replicates of all infected conditions were used in this analysis, with the exception of the 48 h EBOV time point, where a single replicate which failed Quality Control (QC) was excluded from subsequent analysis. Six biological replicates of mock-infected samples were also analysed. Following 14 cycles of amplification, libraries were purified with Ampure XP beads. Libraries were quantified with Qubit and size distribution assessed by AATI Fragment Analyser. These final libraries were pooled in equimolar amounts using the Qubit and Fragment Analyser data. The quantity and quality of each pool was assessed by the Fragment Analyser and, subsequently, by qPCR using the Illumina Library Quantification Kit from Kapa on a Roche Light Cycler LC480II according to the manufacturer’s instructions. The template DNA was denatured according to the protocol described in the Illumina cBot User guide and loaded at 12 pM concentration. To improve sequencing quality control, 1% PhiX was spiked-in. The sequencing was carried out on three lanes of an Illumina HiSeq 2500 with version 4 chemistry, generating 2× 125 bp paired end reads.

### 2.5. Bioinformatics Analysis

Briefly, base calling and de-multiplexing of indexed reads were performed by CASAVA version 1.8.2 (Illumina) to produce 30 samples from the 5 lanes of sequence data, in fastq format. The raw fastq files were trimmed to remove Illumina adapter sequences using Cutadapt version 1.2.1 [34]. The reads were further trimmed to remove low quality bases, using Sickle version 1.200. The reference genome used for alignment was the human reference genome assembly GRCh38, downloaded from Ensembl (ftp://ftp.ensembl.org/pub/release-77/fastahomo_sapiens/dna/Homo_sapiens.GRCh38.dna_sm.primary_assembly.fa.gz). The reference annotation was also downloaded from Ensembl (ftp://ftp.ensembl.org/pub/release-77/gtf/homo_sapiens/Homo_sapiens.GRCh38.77.gtf.gz). The annotated file contained 63,152 genes. R1/R2 read pairs were mapped to the reference sequence using TopHat2 version 2.1.0 [35], which calls the mapper Bowtie2 version 2.0.10 [36]. 

### 2.6. Differential Gene Expression Analysis and Functional Analysis

Mapped reads were further analysed using EdgeR v. 3.3 [37] to calculate normalised counts per million (CPM) and identify differentially expressed genes, comparing infected conditions with mock-infected datasets, as well as infected conditions with each other. Separate time points were amalgamated into a common gene, containing only the most significant differentially expressed genes, reducing this to the 72 h post-infection time point only, when most differentially expressed genes were identified. Correlation, heatmaps, and PCA analysis plots were created in the R environment. Ingenuity Pathway Analysis was used to perform gene ontology analysis and pathway analysis. oPOSSUM version 3.0 [38] was used for Single-Site Analysis with the human reference transcriptomic database to identify significant transcription factors. 

### 2.7. NanoLC MS ESI MS/MS Analysis

Peptides were processed by in-gel LysC digestion and analysed by on-line nanoflow LC using the Ultimate 3000 nano system (Dionex/Thermo Fisher Scientific) coupled with a Q-Exactive mass spectrometer (Thermo Fisher Scientific). Samples were loaded on a Nano-Trap column (Acclaim® PepMap 100, 2 cm × 75 μm, C18, 3 µm, 100 Å), then eluted in-line with the analytical column (Easy-Spray PepMap® RSLC, 50 cm × 75 µm, packed with 2 µm C18, 100 Å particles), fused to a silica nano-electrospray emitter (Dionex, Sunnyvale, United States).

The column was operated at a constant temperature of 35 °C. Chromatography was performed with a buffer system consisting of 0.1% (*v*/*v*) formic acid (buffer A) and 80% acetonitrile in 0.1% formic acid (*v*/*v*) (buffer B). The peptides were separated by a linear gradient of 3.8–50% buffer B over 90 min at a flow rate of 300 nL/min. The Q-Exactive was operated in data-dependent mode with survey and MS/MS scans acquired at a resolution of 70,000 and 17,500, respectively. Up to the top 10 most abundant isotope patterns with charge states +2, +3, and/or +4 from the survey scan were selected with an isolation window of 2.0 Th and fragmented by higher energy collisional dissociation with normalised collision energies of 30. The maximum ion injection times for the survey scan and the MS/MS scans were 250 and 50 ms, respectively, and the ion target value was set to 1E6 for survey scans and 1E5 for the MS/MS scans. 

### 2.8. Protein Identification and Quantification

MS data analysis was processed with MaxQuant software (version 1.5.5.1 [39]) with its internal search engine Andromeda [40]. All settings, except where highlighted below, were set as default. The search included variable modifications of methionine oxidation and N-terminal acetylation, and fixed modification of carbamidomethyl cysteine. Enzyme specificity was set to LysC, minimal peptide length was set to 7 amino acids, and a maximum of two mis-cleavages was allowed. The false discovery rate (FDR) was set to 0.01 for peptide and protein identifications. Quantification was based on Light (Lys 0) and Heavy (Lys 6) SILAC labels. The Andromeda search engine was configured for a database containing Human, EBOV, and RESTV proteins (Uniprot release-2016_10, 21480 entries [41]). The software further included a decoy database, as well as a common contaminants database, to determine the false discovery rate and to exclude false positive hits due to contamination by proteins from different species. The presented protein ratios represent the median of the raw measured peptide ratios for each protein.

### 2.9. ELISArray

Peptide ELISArrays (Qiagen) were utilised to determine protein secretion from infected differentiated THP-1 cells, mock-infected cells, and cells treated with LPS, incubated under identical conditions. The ELISArray for Antiviral Response included the following protein targets: TNF, interleukin (IL)1B, IL6, IL12, IL17, IL8, monocyte chemoattractant protein (MCP)-1, RANTES, interferon-gamma induced protein (IP)10, monokine induced by interferon-gamma (MIG), thymus and activation regulated chemokine (TARC), and interferon (IFN)α. The ELISArray was performed according to the manufacturer’s instructions in triplicate, and resultant optical density readings collected on a spectrophotometer at 450 nm (Molecular Devices). 

### 2.10. Statistical Analysis

qRT-PCR data was analysed using Repeated Measures General Linear Models (GLMs), utilising Bonferonni’s correction. This analysis considered the effect of virus and time on measurements. GLMs were used for statistical analysis of transcriptomic data and proteomic data. Data were tested for sphericity and where data showed a lack of sphericity (*p* < 0.001), the Greenhouse–Geisser correction was applied. False-discovery rates (FDRs) were calculated to assess significance of fold change in protein and transcript abundance, with thresholds of 0.05 FDR and fold change of >2 selected as threshold values to account for background changes in transcript and protein abundance. Correlations were investigated statistically through calculation of *R*^2^ values and significance by Spearman’s correlation. 

## 3. Results

### 3.1. EBOV, Makona, and RESTV Replicate to Similar Titres in THP-1 Cells

**Figure 1:**
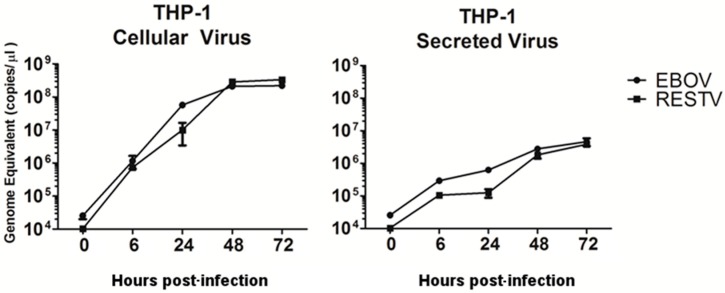
Genome abundance of Ebola virus (EBOV) and Reston virus (RESTV) in THP-1 cells at 0 to 72 h post-infection (hpi) measured in the supernatant, or from 6 to 72 hpi measured in cells using qRT-PCR. The assay was directed to detect mRNA corresponding to the GP gene of either EBOV or RESTV and quantified using the standard curve method with an artificially synthesised RNA transcript control. Data shown are in the log10 scale. Data based on two biological replicates for each condition and PCR performed with three technical replicates per condition for accurate measurement of viral RNA.

RNA-Seq and quantitative proteomics were used to analyse the host response to EBOV and RESTV infection in differentiated THP-1 cells. Duplicate infections were performed at a MOI of 5 for the two different viruses in the THP-1 cells. To assess viral replication in cell culture and the amount of virus released from cells, the abundance of the viral genome was measured using qRT-PCR [4] at 0, 6, 24, 48, and 72 h post-infection (hpi). Viral genome copies were calculated by comparing the qRT-PCR measurements with a synthetically generated control RNA encoding to the GP gene of Makona or RESTV (Figure 1). Statistical analysis using Repeated Measures GLM was performed. The result of this analysis indicated that rates of virus replication were not influenced by virus type at any of the time points tested. There was evidence that there was significant difference between the levels of secreted and cell-associated virus calculated by a paired *T*-test (*p* = 0.046). 

### 3.2. Transcriptomic Analysis

To investigate the abundance of host transcripts in differentiated THP-1 cells infected with either EBOV or RESTV, RNA-Seq analysis was performed with duplicate samples at 24, 48, and 72 h using total RNA prepared from the cell lysate. To establish a background level of variation in cellular transcript abundance, total RNA was prepared from six separate mock-infected controls and sequenced. Non-viral sequence reads were mapped to the human genome. Data was modelled after calculating the negative binomial (NB) distribution. mRNA levels were normalised in order to compensate for sample size factors, and general linear models (GLM) were employed [42]. The trimmed mean M-values (TMM) method was used. Gene counts per million bases (CPM) were calculated using EdgeR in the R environment, and fold change calculated compared to the mock-infected THP-1 cells, or directly comparing EBOV with RESTV-infected THP-1 cells. For EBOV, the analysis indicated that 2085, 1815, and 2849 gene transcripts were increased in abundance and 4115, 2693 and 3902 gene transcripts were decreased in abundance at 24, 48 and 72 hpi, respectively. For RESTV, in total, 1912, 1849 and 1832 gene transcripts were increased in abundance and 3591, 3151, and 2793 gene transcripts were decreased in abundance at 24, 48, and 72 hpi, respectively. 

At 48 h, there was a decrease in the number of transcripts that increased significantly in abundance identified in Makona infection. This decrease was most likely due to greater variation observed between replicates at 48 h for Makona infection, that resulted in a reduced number of transcripts incorporated into the 48 h dataset above the FDR significant threshold. Differences in transcript abundance were investigated by examining the correlation between the datasets corresponding to each condition. The data indicated that host transcript abundance was significantly different in infected cells compared to mock-infected cells, and that host transcript abundance in mock-infected cells clustered closely together, indicating little variability in the control group. To reduce the impact of transcripts that were significantly different between duplicate conditions, these were removed prior to calculation of fold change. Host transcripts with abundant difference of 2 FC or more and identified with an FDR corrected *p*-value of <0.05 were further investigated. 

### 3.3. Verification of RNA-Seq Data Using qRT-PCR

**Figure 2:**
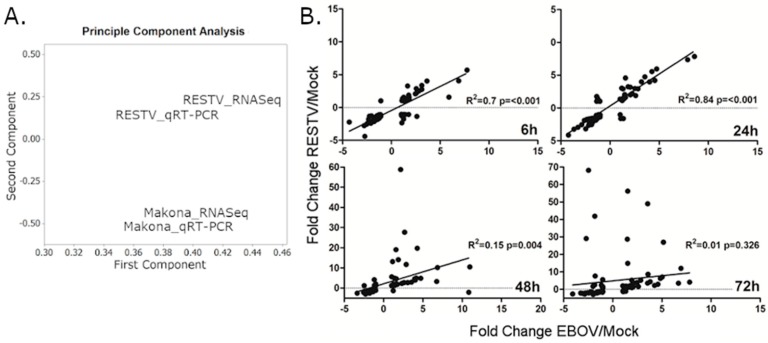
(**A**) Principle component analysis of combined 24, 48, and 72 h data for RNA-Seq and qRT-PCR data, only including RNA-Seq data for transcripts present in qRT-PCR analysis. (**B**) Spearman analysis of the correlation between qRT-PCR data obtained from differentiated THP-1 cells infected with RESTV or EBOV at 6, 24, 48, or 72 h. *R*^2^ values were given to indicate correlation. An R^2^ value of 1 indicated a perfect correlation, and 0 indicated no correlation. Significance testing was performed by Spearman’s correlation without correction, where a *p*-value of <0.05 was likely to be a significant test of correlation. All transcript targets in the qRT-PCR arrays were utilised in this analysis. X axes show fold change in qRT-PCR delta-cT values for EBOV-infected THP-1 cells. Y axes show fold change in qRT-PCR delta-cT values for RESTV-infected THP-1 cells. All data described in this figure were produced from experiments conducted using triplicate infections.

To confirm the RNA-Seq data using a different method, and investigate whether the response was activated at an earlier time point, qRT-PCR was used to measure the abundance of cellular transcripts encoding proteins with antiviral activity at 6, 24, 48, and 72 hpi in THP-1 cells infected with Makona or RESTV in a separate experiment, conducted in triplicate. The qRT-PCR analysis closely resembled the RNA-Seq data for EBOV and RESTV. A Spearman correlation analysis of RNA-Seq and qRT-PCR data showed a significant correlation (ranging from *R*^2^ = 0.74–0.92; *p* ≤ 0.001) for all time points of RESTV and EBOV infection. A principle component analysis was performed, indicating that the relative similarity in qRT-PCR and RNA-Seq data was high (Figure 2A). At the two earlier time points, the transcript abundance for both EBOV and RESTV was closely correlated. At 48 and 72 hpi, the abundance of transcripts involved in antiviral signalling that were measured by qRT-PCR diverged between the two viruses (Figure 2B). 

### 3.4. The Abundance of Interferon-Stimulated Transcripts were Unchanged in Cells Infected with EBOV or RESTV

**Figure 3:**
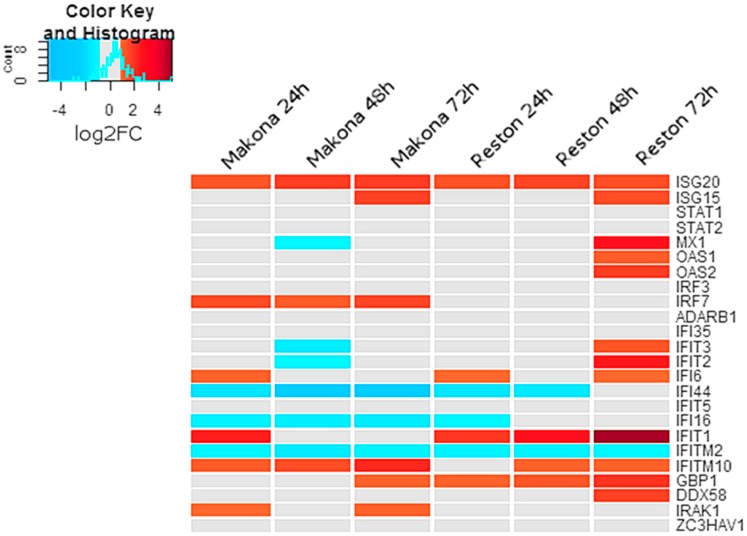
Heatmap showing log2FC in transcript abundance of transcripts encoding genes identified as interferon-stimulated genes (ISG) compared with mock-infected cells. A colour scale bar is provided to aid interpretation, showing change in colour from −5 to +5 with threshold values set at −4 (“Blue”) and +4 (“Red”). A range of −1 to +1 log2FC indicated no change compared with mock-infected cells and is indicated by “Gray”. A histogram is shown in the scale bar, indicating the frequency that genes fall into a particular zone of the colour scale. The histogram indicated that the greatest numbers of transcripts in this heatmap were close to 0 log2FC, with most falling in the range of −2 to +2 log2FC.

RNA-Seq analysis suggested that several transcripts associated with interferon-stimulated genes were significantly increased in abundance in EBOV- or RESTV-infected cells compared to mock-infected cells at 24, 48, and 72 h post-infection (Figure 3). Several interferon-regulated transcripts decreased in abundance in either EBOV or RESTV. MX1 transcripts showed a significant increase in abundance only at 72 hpi in differentiated THP-1 cells infected with RESTV, but not EBOV. Transcripts encoding ISG15 also increased in abundance in both RESTV and EBOV at 72 h, whilst transcripts encoding ISG20 increased in all conditions. IFI44, IFI16, and IFITM2 decreased significantly in abundance in both Makona and RESTV infection. IFIT1 was significantly increased only during infection with RESTV, with the greatest abundance observed at 72 h. 

The activation of interferon-stimulated gene transcripts in EBOV- and RESTV-infected differentiated THP-1 cells was consistent with previous studies investigating gene expression in various model cells lines, including A549 [4], Vero [19], and Huh7 [3] cell lines, as well as several studies of other EBOV variants in primary macrophages [20,22,24,26,27].

### 3.5. The Abundance of Transcripts Encoding Chemokines and Interleukins were Significantly Increased in Cells Infected with Makona

**Figure 4:**
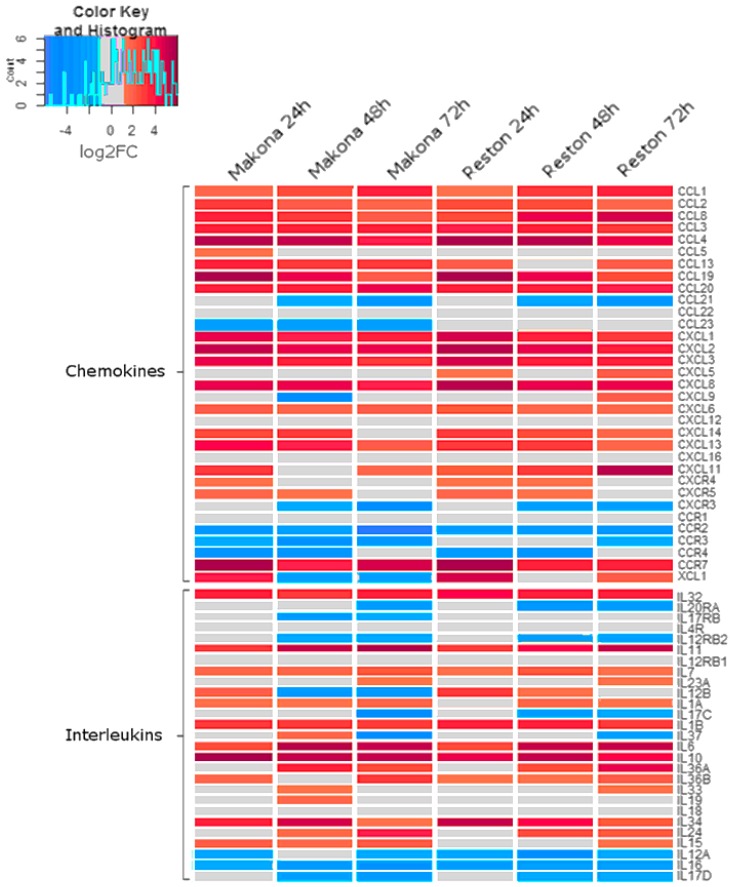
Heatmap showing log2FC in transcript abundance of transcripts encoding genes identified as “chemokines” or “interleukins” compared with mock-infected cells. Five categories of genes are shown, separated by chemokine classification (CC, CXC, CXCR, CCR, and XC); interleukin is shortened to IL for the names of these transcripts. A colour scale bar is provided to aid interpretation, showing change in colour from −6 (“Blue”) to +6 (“Red”). A range of −1 to +1 log2FC indicated no change compared with mock-infected cells and is indicated by “Gray”. A histogram is shown in the scale bar, indicating the frequency that genes fall into a particular zone of the colour scale.

Analysis of patient immune biomarkers in EVD highlighted a variety of chemokines and chemotactic factors which increased to high abundance during acute EBOV infection [7,13,43]. The abundance of transcripts encoding many chemokines was also increased in response to infection with both Makona and RESTV (Figure 4). Transcripts encoding the chemokines CCL2, CCL3 (MIP-1α), IL8, and CXCL13 showed the greatest abundance 24 h after infection with either Makona or RESTV. CXCL11 had the greatest abundance at 24 h in Makona infection, but at 72 h after RESTV infection. CCL5 (RANTES) showed an increase in abundance at 24 h in both EBOV and RESTV infection, but remained unchanged at 48 and 72 h. RANTES is an important chemotactic protein involved in recruitment of lymphocytes to inflammatory sites. CCR1, 2, 3, 4, and CCL21 and 23 were all decreased in abundance in response to Makona and RESTV infection. CCL23, otherwise called myeloid progenitor inhibitory factor (MPIF)-1, is highly chemotactic for resting monocytes and lymphocytes and decreased in abundance in EBOV infection, but not in RESTV infection. 

Pro-inflammatory interleukins, such as IL-1, IL-6, and IL-10, were previously shown to be significantly associated with increased morbidity in human patients [13,43], and were increased in abundance in all time points during Makona infection. Transcripts encoding IL-12B were increased at 24 h, but significantly decreased at 48 and 72 h in Makona infection, whilst they were elevated in RESTV infection at 24 and 48 h. IL-12B is a well described critical inducer of Th1 lymphocytes, critical for antiviral immunity, where absence results in reduced T-cell immune response and increased host susceptibility to infections. Likewise IL-23A, an interleukin with similar function, was decreased at 24 and 48 h in Makona infection and RESTV infection. IL-15 is a potent pro-inflammatory modulator and was raised in all time points during EBOV infection, but did not significantly change in RESTV infection until 72 hpi. The changes in the abundance of cellular transcripts in EBOV- or RESTV-infected cells compared to mock-infected cells may have been caused by differences in activation of upstream transcriptional regulators. 

**Figure 5:**
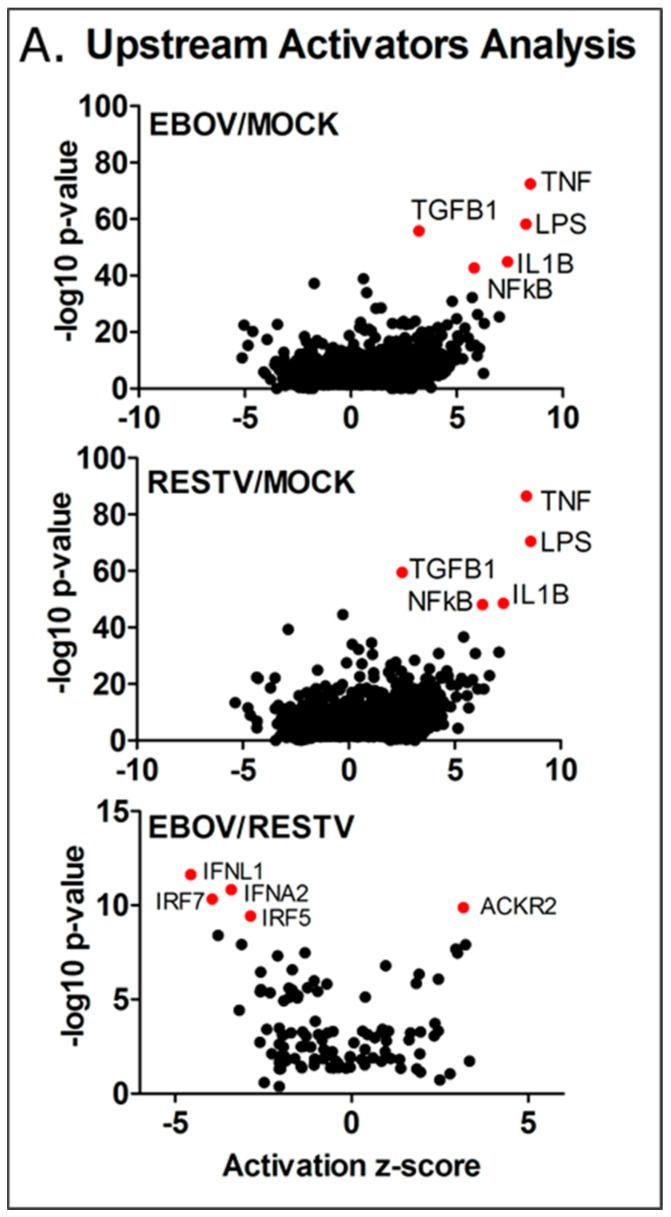
Upstream regulator analysis was performed using canonical pathway analysis data. Transcripts were aligned to canonical pathways and their cumulative increasing or decreasing abundance measurements compared with mock infection, or comparing EBOV to RESTV directly, were used to calculate pathway activation score (z-score). Activation scores range from decreasing activity (−10) to increasing activity (+10) based on predictive calculations. For EBOV/RESTV comparison, the range is smaller (between −5 and +5) due to reduced variation in pathway activity. Only the top five pathways either increasing or decreasing are indicated based on both z-score and −log10 *p*-value. As 72 h represented the most divergent time point comparing EBOV with RESTV, only data collected from this time point were used in these calculations. TNF, LPS (TLR4 ligand), TGFB1, IL1B, and NFκB are shown as highly active upstream regulators in both EBOV and RESTV infection compared with mock infection. Comparing Makona (EBOV) directly with RESTV shows that IFNL1, IFNA2, IRF7, IRF5, and ACKR2 are significant upstream regulators. The direct comparative analysis shows significant though reduced −log10 *p*-values due to reduced numbers of transcripts.

To investigate this, Ingenuity Pathway Analysis was used to group the changes in the abundance of transcripts and identify these potential regulators. The results of this upstream analysis are shown in Figure 5. Z-score was calculated for these regulators, scoring the level of activity proportional to the number of transcripts that significantly changed in abundance that are under their regulatory control. The analysis indicated that NFκB, IL1β, TNF, TGFβ1, and LPS (denoting TLR4 activation) were potential major regulators of gene expression, showing an activity score (z-score) of >2, and a high *p*-value. The observed increase in TNF and IL-1β predicted activity is notable, as these regulators appeared elevated in EBOV Makona infection of A549 cells compared to the Ecran (Mayinga) variant of EBOV from the 1976 outbreak in Zaire [4], and were associated with infection of primary macrophages with the Mayinga variant in previous studies [20,22]. Notably, genes regulated by the FOS and JUN proteins, principal components of the AP-1 pro-inflammatory complex, are over-represented in the data as well, and the activity scores of JUN (EBOV z-score 2.2 and RESTV z-score 2.4) and FOS (EBOV z-score 2.0 and RESTV z-score 2.4) indicated a significantly raised activity score.

### 3.6. Predicting the Activity of Upstream Regulators

Overall, 2669 upstream regulators were identified, common to both EBOV and RESTV infection; 355 significant upstream regulators were unique to EBOV infection, which aligned to several important pathways, including PI3K-Akt, Ras signalling, steroid biosynthesis, and TNF signalling KEGG pathways. ARG1 and NOS1, markers of macrophage activation, were both highly expressed in EBOV, but not RESTV infection. A third comparison highlights the significant differences in upstream regulator predicted activity in EBOV infection compared with RESTV infection. Four of the most significant upstream regulators—those genes showing a significant reduction in activity (2 z-score) and greater than 9 –log10 transformed *p*-value (IFNA2, IFNL1, IRF5, IRF7), and 20 overall in the analysis results—are involved in the interferon response. This correlates with the pathway analysis results, which indicates that interferon regulation was activated in differentiated THP-1 cells infected with either virus. This upstream analysis indicates that several interferon response regulators showed significantly decreased activity in Makona infection compared to RESTV. 

### 3.7. Changes in Gene Expression and Regulation were Reflected at the Protein Level

**Figure 6:**
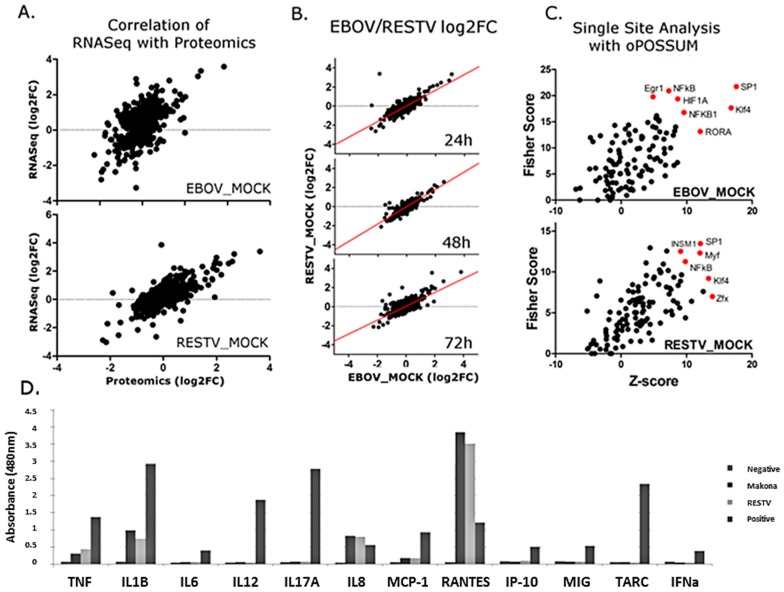
(**A**) Shown is data amalgamated from all three time points assayed (24, 48, and 72 h post-infection). An *R*^2^ value was calculated by Pearson Correlation showing a correlation of 0.85 for RESTV, and a correlation of 0.64 for EBOV (*p* ≤ 0.01); where this value is greater than 0.5, it indicated a positive correlation between these sample datasets. The confidence in this analysis is indicated by *p*-value. Each data point shown illustrates a particular transcript and corresponding protein abundance measurements at 24, 48, or 72 h. (**B**) Separate graphs were shown for protein abundance data acquired from Makona- or RESTV-infected THP-1 cells. (**C**) oPOSSUM upstream analysis demonstrating upstream regulators common to proteins, showing significant changes in abundance at both protein and transcript level. (**D**) Results of a multi-analyte ELISA analysis of supernatant collected from Makona- and RESTV-infected THP-1 derived macrophages at 72 h post-infection. The abundance of the protein identified in supernatant correlates with the optical density measured by a spectrophotometer at 480 nm. Negative control (supernatant only) is shown alongside supernatants from Makona-infected cells, RESTV-infected cells, and positive control (from LPS-stimulated THP-1).

To complement transcriptomic analysis of Makona- and RESTV-infected THP-1 cells, SILAC coupled to LC–MS/MS was used to identify and quantify cellular proteins in both virus-infected and mock-infected cells. Specimens for proteomic analysis were collected from the same cultures used for RNA-Seq data collection. In addition to human proteins identified in the analysis, peptides were identified corresponding to the glycoprotein (GP) and nucleoprotein (NP) from Makona and RESTV. The abundance of peptides from these proteins was not significantly different between the viruses, further verifying that viral levels were similar and comparable. The proteomic data were matched with corresponding transcriptomic (Figure 6A). Most identified proteins did not change significantly (>1 log2 fold change) from those mock-infected, suggesting a global effect on protein production is unlikely. Protein fold change was compared between Makona- and RESTV-infected THP-1 cells, and a plot generated to assess correlation (Figure 6B). This analysis suggests that most proteins correlate between Makona and RESTV. Proteins with corresponding RNA-Seq data were tested with single-site analysis using oPOSSUM. This analysis searches for known transcription factor binding sites upstream of genes of interest. Single-site analysis was plotted in Figure 6C, showing activation (z-score) against significance (fisher score). SP1, NFκB, and Klf4 are common regulators of significant proteins in both Makona and RESTV infection, while HIF1A appears significant only in Makona infection. NFκB was also found to be a significant upstream regulator of differentially expressed transcripts, thus the impact of Makona on NFκB pathway activation has apparent effects on both transcriptional and protein abundance in infected cells. 

To investigate the abundance of secreted proteins under the regulatory control of NFκB pathway activity, a multi-analyte ELISA was performed on cell supernatant collected at 72 h post-infection. Principally, this was aimed at evaluating pro-inflammatory cytokines to assess pathway activity, but also provided an opportunity to assess the levels of interferon being produced during infection, and to compare the impact of Makona and RESTV on cellular cytokine release to that induced by cells treated with bacterial lipopolysaccharide (LPS). LPS is a ligand that binds TLR4, and is a strong inducer of the TLR4-mediated inflammatory response. IL-8 (CXCL8), TNF, IL-1B, and RANTES showed increasing protein concentration commensurate with transcriptomic datasets; this demonstrated that the levels of proteins produced under regulation of NFκB had increased in Makona and RESTV infection. RANTES levels were higher in Makona- and RESTV-infected cells than observed in cells treated with LPS, which correlates with observations from transcriptomic analysis. Interestingly, there was evidence of limited interferon production from cells infected with Makona or RESTV, suggesting that interferon secretion may have been suppressed at a protein level in both Makona and RESTV infection. A small amount of interferon was detected in cells treated with LPS, confirming that interferon production does occur in this cell type (Figure 6D) (Supplementary Materials).

## 4. Discussion

Comparison of ebolaviruses allows identification of unique signatures of viral infection, which in turn can inform design or repurposing of treatments. Based initially on clinical observation, the recently isolated Makona variant of EBOV was suspected of displaying similar disease and replication rates, but differing pathogenesis and virulence compared with previously isolated variants [44]. Analysis of the transcriptomic changes induced by the Makona variant compared with the Ecran (Mayinga) variant (first isolated in 1976) revealed small differences identified in innate immune response pathways [4]. In this study, RNA-Seq was used to measure transcriptomic changes in differentiated THP-1 cells infected with the Makona variant, to attempt to identify signatures of infection in this macrophage-like model cell line and compare data with evidence gathered from previously published studies of other variants of EBOV. IL-1B, IL-6, IL-8, and IL-15 hypersecretion has previously been suggested to correlate with a lethal clinical outcome in human patients infected with EBOV [23]. IL-1B, IL-8, IL-6, and IL-15 showed a significant increase at the transcriptional and protein level in Makona infection of differentiated THP-1 cells. Secretion of these cytokines and chemokines are consistent with stimulation of cells by TNF, and can also be associated with induction via TLR4-mediated activation of the NFκB complex. Differences in the levels of these cytokines may suggest a difference in TNF or NFκB induction by these viruses. Transcripts encoding pro-inflammatory cytokines were highly expressed, and many changed significantly in abundance. This high activity is characteristic of macrophage-like cells when activated. The observed increase in TNF and IL-1β described in THP-1 cells was notable, as these regulators appeared elevated in Makona infection of A549 cells compared to the Ecran (Mayinga) variant of EBOV [4]. IL-6 has previously been described as upregulated in patients and non-human primates infected with Makona and other variants of EBOV [23,43]. 

This strong pro-inflammatory cytokine response was reflected in the analysis of upstream regulators, and was raised in cellular infections with Makona in this study. IPA analysis highlighted NFκB, TNF, IL1β, TGFβ, and LPS (TLR4) as top regulators of significantly changing transcripts. The roles of NFκB and TLR signalling have been highlighted in other studies and were believed to be due to the interaction of viral glycoprotein with TLR4 [26,45,46,47,48]. Single-site analysis using oPOSSUM and proteomic analysis revealed that SP1 and NFκB were major regulators in both Makona and RESTV infection of THP-1 cells, further suggesting a critical role for NFκB. Taken together, proteomics and transcriptomics suggest numerous induced proteins and transcripts were under the regulator control of NFκB.

Similar effects have been observed in influenza A virus infection, requiring NFκB pro-inflammatory activity to effectively infect cells [49,50,51]. Indeed, the host response in primary macrophages to EBOV and RESTV demonstrated significant differences in NFκB activation in a study published by Olejnik et al., describing a study of a different variant of EBOV compared with RESTV [27]. This study deviates from the work described in several ways. Mostly prominently is the case that the viral variant was different, and the methodology used to prepare virus for infection was different. The methodology used by Olejnik et al. [27] had been processed by ultra-centrifugation to purify viral stock. In the case of this study, preparations from amplification cultures on Vero E6 cells were clarified by centrifugation and diluted. This preparation, therefore, had a composition that may be similar to matrices the virus would naturally encounter. It is plausible that our preparation may contain soluble viral and host products, whereas that reported previously [27] may not. This highlights the intriguing possibility that soluble products have a critical role in enhancing viral infection of cells through the activation of cellular inflammatory processes that were shown to be important by Olejnik et al. [27]. Additionally, the lack of transcriptomic and proteomic differences observed between cells infected with Makona and RESTV may be attributable to the use of differentiated THP-1 cells, rather than primary cells. THP-1 cells are known to display a highly inflammatory phenotype upon activation [28,29,30]. Differences between the work described here and previous studies on EBOV in primary macrophages are expected, as immortalised cell lines are known to have atypical interferon signalling. Previous studies of other EBOV variants infecting macrophages have noted a significant induction of inflammatory response [20,22,26,27,33], which is also observed in this study of Makona. The pathogenesis of EVD is known to involve early infection of macrophages and dendritic cells. As both Makona and RESTV elicited similar responses, it may be that the direct pathogenic effects of macrophage infection do not play a significant role in the overall pathogenesis of EVD; however, evidence from this study and patients indicate that macrophage activation likely has indirect impact on disease progression through the hypersecretion of cytokines, and the disturbance of signalling to other immune cells. The infections performed in this study elicited an interferon response only detectable by transcriptomics, but was not observed at the proteomic level. Immunoassay measurement of supernatant showed the strong induction interferon alpha in cells treated with LPS, but not in those uninfected and untreated, or cells that were infected with Makona or RESTV. This is in contrast to reports that the Makona is a strong inducer of interferon production in vivo and in patients; however, there is a known dichotomy between in vivo and in vitro interferon induction observed in EBOV infection. In addition, our studies focused on responses in macrophage-like cells only, whereas in whole animal system, multiple cell types interact with one another and the role of each type cannot be ascertained in great depth.

**Figure 7:**
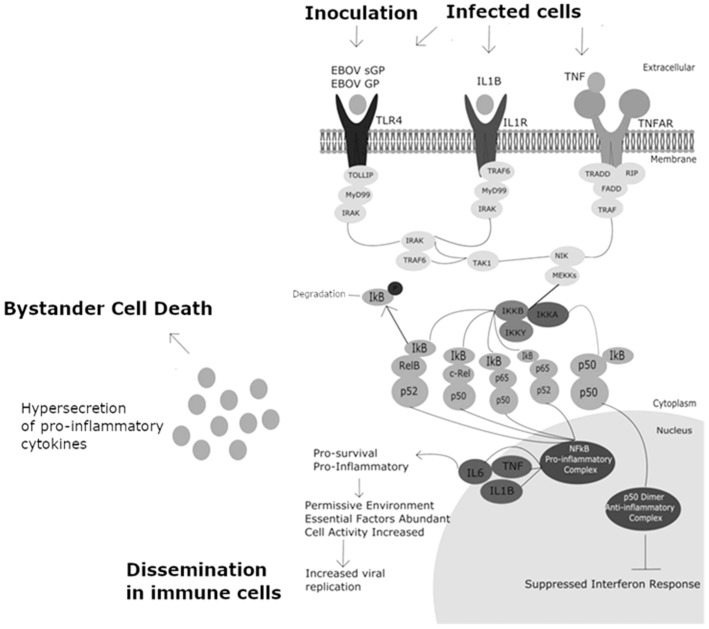
Schematic diagram of the hypothesised model of the relationship between EBOV infection and NFκB activation. Activation occurs via stimulation of multiple receptors, including TLR4 (by EBOV sGP/GP), IL1R by IL1β, and TNFR by TNF. Heterodimeric complexes composed of p65, p50, 052, c-Rel, RelB are pro-inflammatory, homodimers may also form from p50–p50. P50 homodimers are anti-inflammatory and act as a negative feedback system controlling NFκB pro-inflammatory activation. P50 homodimers suppress the interferon response, and the activity of the EBOV proteins VP24 and VP35 further contribute to rendering the type 1 interferon response ineffective. Pro-inflammatory proteins are produced, and NFκB heterodimers result in pro-survival signalling, preventing apoptosis. Increased cellular activity and upregulation of as yet unidentified host factors result in increased viral replication rates, and improved permissiveness for viral infection.

Significant differences in the abundance of transcripts regulated by NFκB in Makona and RESTV infection may suggest an indirect role for NFκB in the lifecycle of ebolaviruses. No direct interactions with NFκB have so far been demonstrated for EBOV or for similar viruses such as dengue or influenza viruses with NFκB. We propose a model illustrating the role of NFκB in ebolavirus lifecycle, which is shown in Figure 7. Transcriptomic and proteomic analyses have identified TNF, IL-1B, TLR4, and growth factor-associated transcripts and proteins which may be part of a potent NFκB-mediated response. In this proposed model, an overstimulation of the NFκB response results in hypersecretion of inflammatory cytokines by affected cells, and contributes to the prevention of cellular apoptosis which has been observed in previous studies [52] by promoting cell survival. Hypersecretion of pro-inflammatory cytokines, meanwhile, has deleterious effects on cells of the adaptive immune response, and may explain the bystander cell death observed affecting lymphocyte populations, which resist infection with EBOV but are susceptible to induced cell death [20,22,23]. The pro-inflammatory p65 component of the NFκB complex was recently shown to be a major contributory element of the observed requirement for NFκB activation to permit influenza A infection, alongside c-Rel co-factor [53]. The high degree of pro-inflammatory signalling downstream of the NFκB complex highlights proteins contributing to NFκB activity as interesting targets for future study. Together, these results indicate that NFκB induction may be important for ebolavirus species.

## 5. Conclusions

The aim of this project was to characterise the host response to the Makona, comparing the profiled transcriptomic and proteomic changes with those induced by infection with the seemingly apathogenic RESTV, helping to identify transcriptomic and proteomic signatures of infection between similar viruses of differing pathogenicity, and contrast observed transcriptomic changes with those seen during infection with other EBOV variants described in the literature. The analyses described in this study illustrated the surprising similarity in the transcriptional profiles found in cells infected with the ebolavirus species tested. Surprising because other recent studies have shown that the inflammatory response, in particular, has notable differences in the strength of induction between infection with other variants of EBOV and RESTV. The main determinants of successful cell infection are likely shared among ebolavirus species of differing pathogenicity, and the host factors related to this are under the regulatory control of the NFκB complex. Maintaining a potent NFκB-mediated inflammatory response may be critical in the ebolavirus lifecycle, and as well as being a determinant of successful infection by ebolaviruses, overstimulation of the inflammatory response may have a more deleterious effect in man—potentially contributing to divergent clinical phenotypes observed between EBOV variants such as Makona and RESTV. The data described in this study show that infection of differentiated THP-1 cells with Makona causes similar effects to those elicited by infection with RESTV in this cell model. The observed transcriptomic and proteomic changes induced by Makona are similar to other variants of EBOV studied previously, and the Makona variant does not appear to display unique properties of infection. 

## Supplementary Materials

The following are available online at https://www.mdpi.com/1999-4915/11/11/1023/s1: Supplementary Data A: Supplementary data are provided as an excel table of all transcriptomic data produced using EdgeR in the R environment from data collected in experiments utilising the Ebola virus Makona variant and Reston virus; Supplementary Data B: Supplementary data are provided as an excel table of all proteomic data produced using Mascot and Andromeda in collected in SILAC-Proteomic experiments utilising the Ebola virus Makona variant and Reston virus.
